# Wnt1 Accelerates an *Ex Vivo* Expansion of Human Cord Blood CD34^+^CD38^−^ Cells

**DOI:** 10.1155/2013/909812

**Published:** 2013-08-20

**Authors:** Kamonnaree Chotinantakul, Patcharee Prasajak, Wilairat Leeanansaksiri

**Affiliations:** ^1^Stem Cell Therapy and Transplantation Research Group, Suranaree University of Technology, Nakhon Ratchasima 30000, Thailand; ^2^School of Microbiology, Institute of Science, Suranaree University of Technology, Nakhon Ratchasima 30000, Thailand

## Abstract

Cord blood hematopoietic stem cells (CB-HSCs) transplantation has been increasing gradually with facing the limitation of insufficient quantity of HSCs in each CB unit. Therefore, efficient expansion methods which can maintain stem cell characteristics are needed. In this study, umbilical CB-CD34^+^ cells were cultured in two different cytokine cocktails: 4 factors (4F = Flt3-L, SCF, IL-6, and TPO) and 5 factors (5F = Wnt1 + 4F) in both serum and serum-free media. The data revealed that the best condition to accelerate an expansion of CD34^+^CD38^−^ cells was serum-free culture condition supplemented with 5F (5F KSR). This condition yielded 24.3 ± 2.1 folds increase of CD34^+^CD38^−^ cells. The expanded cells exhibited CD34^+^ CD38^−^ CD133^+^ CD71^low^ CD33^low^ CD3^−^ CD19^−^ markers, expressed *nanog, oct3/4, c-myc,* and *sox2* genes, and maintained differentiation potential into lymphoid, erythroid and myeloid lineages. The achievement of CD34^+^CD38^−^ cells expansion may overcome an insufficient quantity of the cells leading to the improvement of the stem cell transplantation. Altogether, our findings highlight the role of Wnt1 and the new culture condition in stimulating hematopoietic stem/progenitor cells expansion which may offer a new therapeutic avenue for cord blood transplantation, regenerative medicine, stem cell bank applications, and other clinical applications in the future.

## 1. Introduction

Hematopoietic stem cells (HSCs, CD34^+^CD38^−^) obtained from umbilical cord blood (UCB) have been studied extensively in stem cell research for advanced cellular therapies [1]. Cord blood (CB) contains HSCs expressing low immunogenicity which render CB to be the promised source of stem cells for transplantation [2]. Moreover, CB transplantation displays advantages over bone marrow and mobilized peripheral blood transplantations in the aspects of noninvasive collect procedure, richness in hematopoietic stem/progenitor content [3], and lower incidence of acute graft-versus-host disease [4]. However, the quantity of HSCs is limited in a single CB unit and may raise the risk of engraftment failure, especially in the adult transplantation [5]. Thus, an increase in HSCs population without changing in their phenotype and losing their repopulating ability is required for successful clinical transplantation. These obstacles, therefore, constitute a challenge to researchers to overcome.

The majority of publications aim to expand CD34^+^ cells rather than CD34^+^CD38^−^ cells expansion. However, CD34^+^CD38^−^ cells indeed conserve more primitive HSCs population, which contain more efficiency to reconstitute all blood cell types *in vivo*. The CD34^+^CD38^−^ cells also contain the plasticity of differentiation into many cell types *in vitro* such as adipocytes [6], brain cells (neurons and astrocytes) [7], cardiomyocytes [8], liver cells [9], myoblasts [10], myoendothelial [11], osteochondrocytes [12], and pancreatic cells [13]. The CD34^+^ populations from bone marrow and cord blood are heterogeneous and contain both CD34^+^CD38^−^ and CD34^+^CD38^+^ fractions. There is approximately 0.05% ± 0.08% of the mononuclear cells present in cord blood which are CD34^+^CD38^−^ cells. In isolated CD34^+^ population, about 1–10% was found to be the primitive CD34^+^CD38^−^ cells that were quiescent and contained long-term culture-initiating cells (LTC-ICs) which were able to generate colony-forming unit cells (CFU-C) [14]. Moreover, SCID-repopulating cells (SRCs) were found only in the CD34^+^CD38^−^ fraction while CD34^+^CD38^+^ fraction could not be engrafted in NOD/SCID mice [15]. Mishima and colleagues succeeded to expand CB-CD34^+^CD38^−^ cells with approximately 7-fold increase by culturing the cells with osteoblast-differentiated MSC feeder cells supplemented with SCF, TPO, Flt3-L, IL-3, and IL-6 [16]. However, this procedure is complicate to handle, inconvenient to perform a large-scale culture, and HSCs may attach to the feeder cells.

The Wnt signaling proteins play key roles during the early development of embryo and in adult tissue homeostasis. Wnt signaling also regulates embryonic stem cells (ESCs) differentiation and supports the maintenance of self-renewal of ESCs [17]. Several studies have shown the role of Wnt family in the regulation of HSCs stemness and self-renewal capacity. Wnt1/*β*-catenin signaling has been reported to mediate BMP-4-induced self-renewal in mouse ESCs [18]. Mice deficient in 12/15-lipoxygenase- (12/15-LOX-)mediated unsaturated fatty acid metabolism represented a lower number of long-term HSCs with a reduction in canonical Wnt signaling [19]. Recently, Wnt/*β*-catenin-activated mesenchymal stem cells (MSCs), that provide an activated niche, have been shown to promote self-renewal of HSCs with about 4.5 folds in irradiated mice bone marrow [20]. It has been reported that Wnt3a protein implicates in signaling to stimulate not only self-renewal of HSCs but also cell fate decision during hematopoiesis [21]. Nikolova and colleagues indicated that Wnt1 and Wnt3a conditioned medium (CM) were capable to enhance the proliferation and preserve immature state of CB-CD133^+^, while WNT4, WNT5a, and WNT11-CM have been shown to promote nonhematopoietic differentiation [22]. However, the role of Wnt1 in HSCs expansion has not yet been explored. In this work, we demonstrated for the first time that Wnt1 supplementation in the cytokines-based serum-free culture for 7 days could significantly enhance the proliferation of CB-CD34^+^CD38^−^ and CB-CD133^+^CD38^−^ cells. Both populations have been known to contain self-renewal capacity. In addition, the expanded cells exhibited low differentiated cells, maintained hematopoietic stem, and progenitor cells (HSPCs) properties to differentiate into all blood cell types *in vitro*.

## 2. Materials and Methods 

### 2.1. Purification of Human CB-Derived CD34^+^ Cells

Human UCB samples were obtained from umbilical cord after the delivery of normal pregnancies with inform of consent. The research has been carried out in accordance with the approval of ethics committee for researches involving human subjects of Suranaree University of Technology, based on the Declaration of Helsinki of the World Medical Association. CB samples were processed within 4 hours. Mononuclear cells (MNCs) were isolated by Ficoll-Hypaque density gradient centrifugation (1.077 g/L, GE Healthcare Bio-Sciences B, Sweden). Then, CB-CD34^+^ cells were purified from MNCs by Dynabeads M-450 and DETACHaBEAD CD34 as manufacturer's instruction (Dynal AS, Norway).

### 2.2. Cell Culture and Expansion

Isolated CD34^+^ cells were cultured in Iscove's Modified Dulbecco's Medium (IMDM; Gibco, CA, USA) supplemented with either 10% fetal bovine serum (FBS) or KnockOut Serum Replacement (KSR; Invitrogen, CA, USA) and the combination of human recombinant cytokine cocktails (Peprotech, Rocky Hill, NJ, USA). The culture was separated into 4 groups: (1) 4 factors in cIMDM (4F cIMDM): IMDM + 10% FBS + Flt3-ligand (Flt3-L, 100 ng/mL), stem cell factor (SCF, 100 ng/mL), interleukin-6 (IL-6, 100 ng/mL), and thrombopoietin (TPO, 10 ng/mL); (2) 5F in cIMDM (5F cIMDM): IMDM + 10% FBS + Wnt1 (20 ng/mL) + Flt3-L (100 ng/mL), SCF (100 ng/mL), IL-6 (100 ng/mL), and TPO (10 ng/mL); (3) 4 factors in serum-free medium (4F KSR): IMDM + 10% KSR + Flt3-L (100 ng/mL), SCF (100 ng/mL), IL-6 (100 ng/mL), and TPO (10 ng/mL); (4) 5F in serum-free medium (5F KSR): IMDM + 10% KSR + Wnt1 (20 ng/mL) + Flt3-L (100 ng/mL), SCF (100 ng/mL), IL-6 (100 ng/mL), and TPO (10 ng/mL). All experiments were cultured at 37°C in 5% O_2_, 5% CO_2_ for 7 days. The expanded cells were enumerated every day by hemocytometer. After 5 days of expansion, cells were subjected to liquid culture differentiation and colony-forming cell assay as below. Moreover, on day 5 and day 7 of expansion, cells were characterized by the expression of CD3, CD19, CD33, CD34, CD38, CD71, and CD133 cell surface molecules by flow cytometry analysis. 

### 2.3. Proliferation Assay

CD34^+^ cells were cultured in 96-well plates in 4F cIMDM, 4FW cIMDM, 4F KSR, and 4FW KSR mediums (1 × 10^4^ cells/well). After culture for 3, 5, and 7 days, cell proliferation was measured by 5-bromo-2′-deoxyuridine (BrdU) incorporation for 24 h with a commercial BrdU cell proliferation kit (Calbiochem, Germany) according to the manufacturer's protocol. Briefly, 20 *μ*L BrdU solution (1 : 2000) was added into each well. After the 96-well dish was incubated for 24 h, the dish was centrifuged at 1000 rpm for 10 min. Then, the contents were removed. The 200 *μ*L of fixative/denaturing solution was added into each well and incubated for 30 min. Next, 100 *μ*L of 100x anti-BrdU antibody (1 : 100) was mixed and incubated for 1 h. Cells were washed and mixed with 100 *μ*L of peroxidase goat anti-mouse IgG horse radish peroxidase conjugate. After the plate was incubated for 30 min, cells were washed and added to 100 *μ*L of the substrate solution. The proportion of incorporated BrdU was then determined by measuring the absorbance at dual wavelengths of 450–540 nm (xMark Microplate Absorbance Spectrophotometer, Biorad, USA).

### 2.4. Flow Cytometric Analysis

Phenotypic analysis of the expanded CD34^+^ cells was performed on day 5 and day 7 of the culture including the expression of cell surface markers CD3, CD19, CD33, CD34, CD38, CD71 (BD pharmingen, San Jose, CA, USA), and CD133 (MACS, Miltenyi Biotec, Germany). Expanded CD34^+^ cells from each experiment were collected and resuspended in PBS. After labeling the cell suspension with monoclonal antibodies, the cells were incubated for 30 min on ice. The mixtures were then washed by PBS to remove excess antibodies and followed by fixing the cells with 4% paraformaldehyde. Cells were subjected for surface markers investigation by flow cytometry analysis using CellQuest Pro software (FACSCalibur, Becton Dickinson, San Jose, CA, USA). The number of each subpopulation at an indicated time point was normalized from mean total number of nucleated cells and the mean percentage of positive cells in each subpopulation. The fold increase in expansion was calculated by dividing the total cell number in each population by the number of subpopulation at starting culture.

### 2.5. Liquid Culture Assay

The expanded CD34^+^ cells were subjected to liquid culture assays for all 3 hematologic cell lineages differentiation: myeloid, lymphoid, and erythroid lineages. Cells were cultured in IMDM supplemented with 10% FBS and the combination of recombinant human cytokines (Peprotech, Rocky Hill, NJ, USA) as follows: (a) erythrocytic lineage; 20 ng/mL EPO and 100 ng/mL SCF, (b) megakaryocytic lineage; 100 ng/mL TPO and 100 ng/mL SCF, (c) granulocyte and macrophage lineages; 20 ng/mL GM-CSF and 30 ng/mL IL-3, (d) mast cell and granulocyte lineages; 100 ng/mL SCF and 30 ng/mL IL-3, and (e) lymphoid lineage; 100 ng/mL SCF, 100 ng/mL Flt3-L and 50 ng/mL IL-7. The OP9 cells were used as feeder cells for lymphoid differentiation. The cells were cultured in a humidified 5% O_2_, 5% CO_2_ atmosphere at 37°C for 14–30 days. Cytospins of each culture were prepared and subjected to Wright-Giemsa staining. Image analysis was visualized by microscope (Olympus BX51, Olympus, Japan) and captured by a digital CCD camera (Olympus DP72, Olympus, Japan).

### 2.6. Colony-Forming Cell (CFC) Assay

The capacity of expanded CD34^+^-enriched-population to generate hematopoietic clonogenic progenitors was analyzed. Briefly, the cells were harvested after 5 days of expansion process and then cultured in MethoCult H4434 for CFC assay as per the manufacturer procedures (Stemcell Technologies, Vancouver, BC, Canada). All cells were cultured in 35 mm culture dishes for 14 days at 37°C with 5% O_2_, 5% CO_2_ and 95% humidity. CFCs were then scored on day 14 according to their morphology [25] under inverted microscope (CKX41, Olympus, Japan) and the photographs were captured by a digital CCD camera (Olympus DP72, Olympus, Japan). Fresh isolated CD34^+^ cells controls were included.

### 2.7. Gene Expression Analysis by Real-Time RT-PC

Total RNA (700 ng) and cDNA of each sample were prepared using RNA minikit (Geneaid, Taiwan) and Superscript First-Strand Synthesis System (Invitrogen, USA) as per manufacturer's instructions, respectively. Power SYBR PCR mix contained 1x SYBR green PCR master mix, 200 nM for *oct3/4*, *nanog,* and *GAPDH*, 300 nM for *c-myc* and *sox2* primers and 1.5 *μ*L of cDNA. The primer, sequences used in the real-time RT-PCR analysis are listed in Table 1. The reaction was performed for 10 minutes at 95°C, 40–50 cycles of 15 s at 95°C, and 1 minute at the 60°C followed by dissociation step (ABI 7900HT Fast Real-Time PCR System, Applied Biosystems). The assay was performed in triplicate with each template and the negative control. Relative quantification of gene expression was performed using Applied Biosystems Sequence Detection software v.1.2.2.

### 2.8. Statistical Analysis

Data were presented as the mean values ± the standard deviation. Statistics were calculated with SPSS software (SPSS Inc., Chicago, IL, USA). Results were analyzed with one-way ANOVA test followed by Tukey's HSD test. Values of *P* < 0.05 were considered statistically significant.

## 3. Results

### 3.1. Wnt1 Enhances Hematopoietic Stem/Progenitor Cells (HSPCs) Proliferation

**Figure 1:**
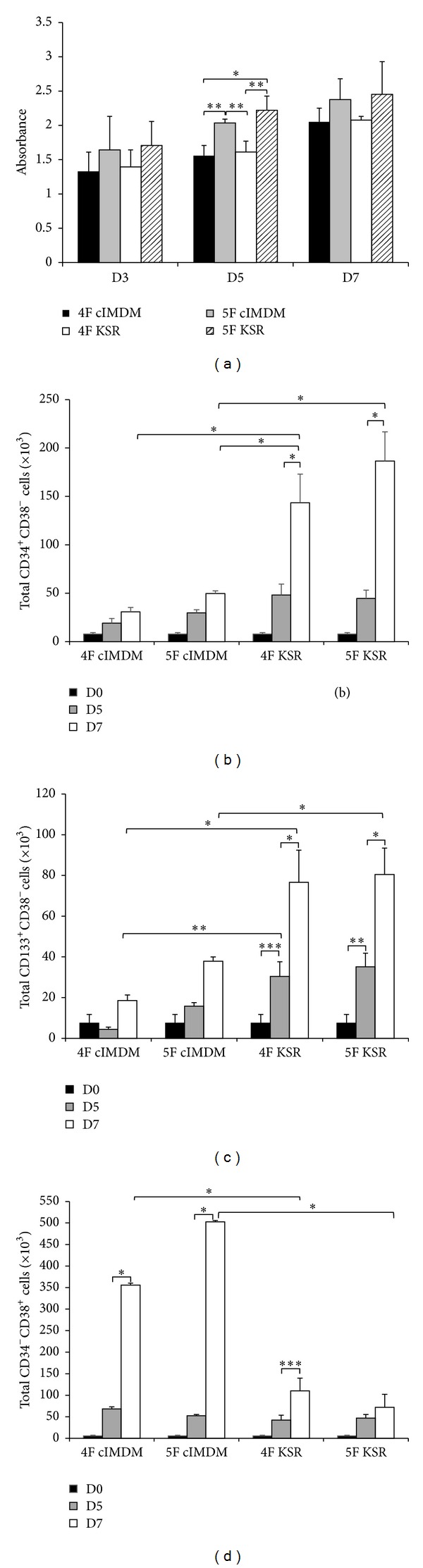
Proliferation of expanded CB cells. CB-CD34^+^ were maintained in 4 different cultures media: 2 serum-containing media (4F cIMDM, 5F cIMDM) and 2 serum-free media (4F KSR, 5F KSR). (a) Proliferation assay, (b) growth profile of total CD34^+^CD38^−^ cells, (c) CD133^+^CD38^−^ cells, and (d) CD34^−^CD38^+^ cells. After culturing the cells for 5 and 7 days, cells were harvested for analysis and normalized with day 0 (*n* = 3). *P < 0.001, **P < 0.02, and ***P < 0.05.

Human CB-CD34^+^ cells were separated into 4 groups: 4F cIMDM, 5F cIMDM, 4F KSR, and 5F KSR. All cultures were incubated at 37°C, 5% O_2_, 5% CO_2_, and 95% humidity for 7 days. To assess whether various culture conditions could accelerate cells proliferation and preserve hematopoietic stem cells phenotype throughout the culture period, we analyzed the proliferation and fold increase of the cells after expansion. Here, our results showed that the proliferation rate of total nucleated cells in 5F KSR medium displayed the highest rate on both day 5 and day 7 of the cultures (Figure 1(a)). The proliferation rate was significantly higher in 5F KSR (2.2 ± 0.2) compared to 4F KSR (1.6 ± 0.2) and 4F cIMDM (1.6 ± 0.2) at day 5 (*P* < 0.02). In addition, the expansion of expanded cells in 5F cIMDM culture (2.0 ± 0.1) was significant higher than that of 4F cIMDM medium (1.6 ± 0.2) at day 5 (*P* < 0.02) but not insignificant at day 7 of cultures (Figure 1(a)). Then, we investigated cell surface markers of the HSPCs in expanded cells by staining cells with CD34, CD38, and CD133. The CD34^+^CD38^−^ cells represent primitive HSC population which is very crucial for therapeutic purpose. There was a slightly increase of CD34^+^CD38^−^ population in the expanded cells cultured in 4F cIMDM and 5F cIMDM at day 5 as 2.6 ± 1.1 and 3.9 ± 1.0 folds compared with day 0 of the control cells, respectively (Figure 1(b) and Table 2). These cells continuously expanded more over time as 4.2 ± 1.3 and 6.6 ± 1.4 folds at day 7 of expansion process, respectively. 

**Figure 2:**
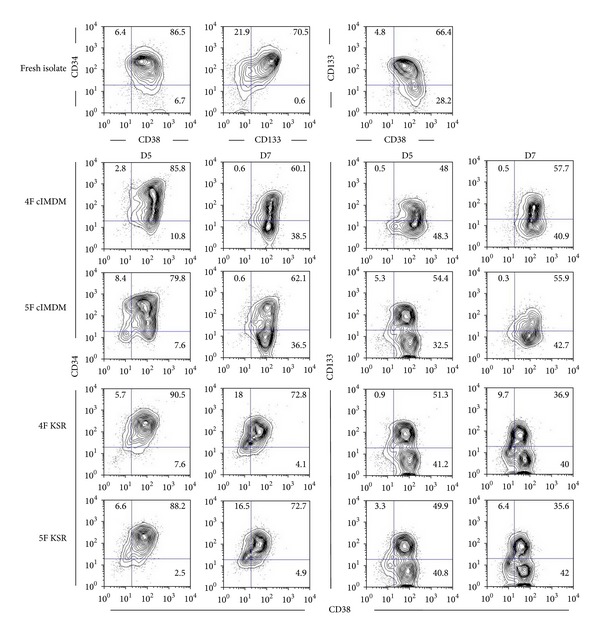
Immunophenotype of expanded cells. Representative cell surface markers CD34/CD38/CD133 of fresh isolated CD34^+^ cells and expanded cells at 5 and 7 days of each culture condition.

Interestingly, cells that maintained in 4F KSR and 5F KSR for 7 days revealed the significant multiplication of CD34^+^CD38^−^ population as 18.5 ± 0.4 and 24.3 ± 2.1 folds, respectively (*P* < 0.001 versus 4F cIMDM and 5F cIMDM; Figure 1(b) and Table 2). The representative flow cytometry analyses of expanded cells collected on day 5 and day 7 are shown in Figure 2, and the immunophenotypic of each subpopulation (CD34^+^CD38^−^, CD133^+^CD38^−^, and CD34^+^CD38^−^ cells) of expanded cells characterized at day 5 and day 7 is summarized in Table 3. These data suggested that expansion of the cells in serum-free medium containing cytokines cocktail of 4F or 5F can serve as great culture conditions for CD34^+^CD38^−^ cells expansion. In addition, in the presence of Wnt1, this cytokine exhibited the most potent acceleration effect on CD34^+^CD38^−^ cells proliferation. Moreover, cells cultured in 4F KSR and 5F KSR conditions contain less CD34^−^CD38^+^ cells (differentiated cells; 20.1 ± 6.8 and 13.0 ± 3.7 folds, resp.) than those cells cultured in 4F cIMDM and 5F cIMDM (62.8 ± 5.0 and 89.7 ± 12.2 folds, resp.) as observed at day 7 (*P* ≤ 0.001; Figure 1(d) and Table 2). This data indicated that 4F KSR and 5F KSR conditions could preserve HSCs phenotype of the cells better than serum-containing medium. 

Surprisingly, culture condition of 4F KSR and 5F KSR also revealed the significant enhancement of CD133^+^CD38^−^ subpopulation proliferation as 11.5 ± 3.4 and 12.3 ± 4.0 at day 7 of cultures, respectively (*P* < 0.05 versus 4F cIMDM; Figure 1(b) and Table 1). These cells also contain blood cells repopulating capacity *in vivo* [26, 27]. Altogether, our findings suggest that 4F KSR and 5F KSR can augment CD133^+^CD38^−^ subpopulation proliferation along with CD34^+^CD38^−^ cells' expansion. The advantage of the presence of both populations simultaneously is that they can synergize and enhance the capacity of mature blood cells reconstitution which will benefit the cells transplantation. In addition, cells cultured in serum-free medium could maintain their stemness than in the presence of serum. Thus, these data demonstrate that Wnt1 is a potent stimulator of CD34^+^CD38^−^ and CD133^+^CD38^−^ cells' proliferations. 

### 3.2. Phenotypes of Expanded Cells

**Figure 3:**
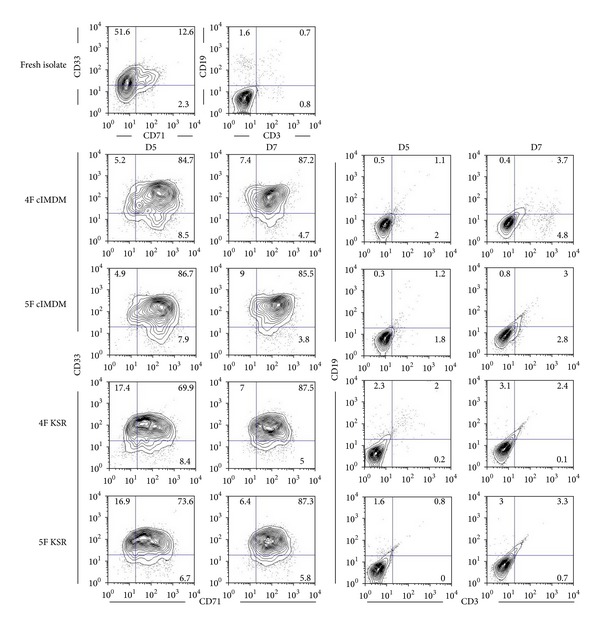
Characterization of progenitor cells. Representative cell surface markers CD33/CD71 and CD3/CD19 of fresh isolated CD34^+^ cells and expanded cells at 5 and 7 days of each culture condition.

As we demonstrated that all 4 culture conditions contained CD34^+^CD38^−^, CD133^+^CD38^−^, and CD34^−^CD38^+^ populations. We next further investigated more phenotype of progenitor cells in these expanded cells. To this end, cells from 4 conditions were separately harvested and subjected for myeloid, lymphoid, and erythroid lineages analysis using CD33 (myeloid), CD71 (erythroid), CD3, and CD19 (lymphoid) markers. The results indicated the significant presence of early markers of myeloid/erythroid progenitors but not lymphoid progenitors in all culture conditions (Figure 3). However, cells cultured in 4F cIMDM and 5F cIMDM contained more myeloid/erythroid progenitors than cells cultured in both 4F KSR and 5F KSR. In addition, the results demonstrated that medium containing serum induced alteration of cell phenotypes by the loss of CD34^+^ cells and obtained more committed progenitors CD34^−^CD38^+^ population (4F cIMDM = 62.8 folds and 5F cIMDM = 89.7 folds) than those observed in serum-free medium (4F KSR = 20.1 folds and 5F KSR = 13.0 folds; Table 2). This data suggests serum effect in enhancement of spontaneous differentiation.

Taken together, CB-CD34^+^-enriched populations cultured in 4F KSR and 5F KSR exhibit (1) higher significant increase of CD34^+^CD38^−^ and CD133^+^CD38^−^ cells than those in 4F cIMDM or 5F cIMDM, (2) more efficiently maintain CD34^+^ phenotype than 4F cIMDM or 5F cIMDM conditions, (3) less myeloid/erythroid progenitors than medium-containing serum conditions. Interestingly, all 4 conditions show the same profile of lymphoid lineage with no significant presence of these lineage progenitors. Therefore, 4F KSR and 5F KSR can expand and maintain stemness of CD34^+^ cells more than 4F cIMDM or 5F cIMDM conditions. 

### 3.3. Expanded Cells Can Reconstitute Blood Cell Lineages

**Figure 4:**
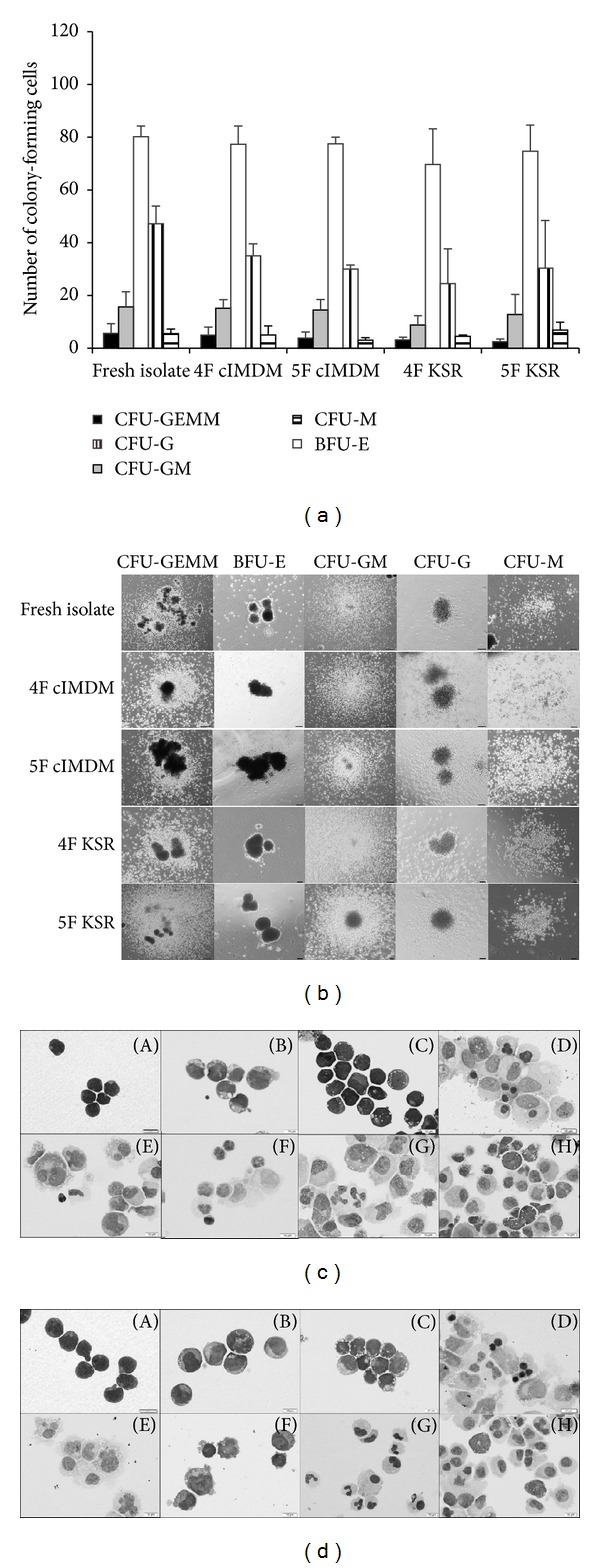
Hematopoietic colony-forming units and differentiation capacity of expanded CB-CD34^+^ cells *in vitro*. (a) Colony-forming assay of 5-day-enriched CB-CD34^+^ cells in 4 different culture conditions (mean ± SD; *n* = 3). (b) Photographs of CFU-GEMM, CFU-GM, BFU-E, CFU-G, and CFU-M after incubated in methylcellulose for 14 days. Liquid differentiation assay of 5-day-expanded CB-CD34^+^ cells from (c) 4F KSR and (d) 5F KSR conditions. In (c) and (d), (A) fresh CB-CD34^+^ cells, (B) 5-day-expanded CB-CD34^+^ cells, (C) 7-day-expanded CB-CD34^+^ cells, (D) erythroid lineage differentiation (EPO+SCF), (E) megakaryocytes differentiation (TPO+SCF), (F) lymphoid lineage differentiation (IL-7, Flt3-L, SCF+OP9), (G) granulocytes and macrophage lineages differentiation (GM-CSF+IL-3), and (H) mast cells and granulocyte lineages differentiation (SCF+IL-3). Bar correspondences as 10 *μ*m.

The colony formation *in vitro* indicates the efficiency of hematopoietic stem/progenitor cells to develop into myeloid and erythroid colonies in the presence of various combinations of cytokine factors. In this regard, this capacity was analyzed in the expanded CD34^+^ cells. After expanded CD34^+^ cells for 5 days in 4 different conditions, cells were harvested and seeded in semisolid methylcellulose as described in Materials and Methods. The results showed that expanded cells in all 4 various culture conditions contained the ability to produce clonogenic progenitor cells: CFU-GEMM, CFU-GM, BFU-E, and CFU-M similar to the fresh isolated CD34^+^-cells (Figures 4(a) and 4(b)). Although the total CFU-G number in all cytokine culture conditions showed a slightly decrease in the number as compared with fresh isolated group, it was statistically insignificant. In addition, expanded CD34^+^ enriched population in 4F KSR and 5F KSR could generate more mature blood cells in liquid culture differentiation (Figures 4(c) and 4(d)) as well as those cultured in cIMDM conditions (data not shown). These findings indicated the achievement of repopulating capacity of expanded HSPCs population *in vitro*. 

### 3.4. Expanded CD34^+^-Enriched Population in 4F KSR and 5F KSR Preserve Pluripotency Genes Expression

**Figure 5:**
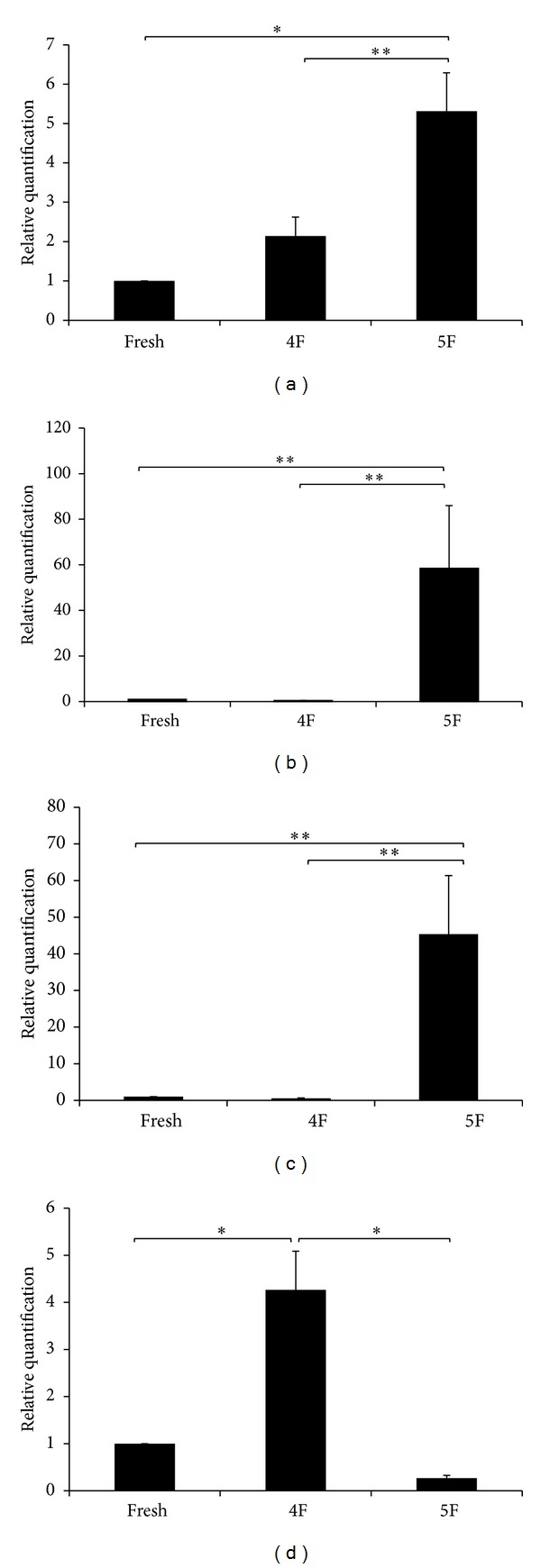
Pluripotency genes expression. Relative quantitation of (a) *c-myc*, (b) *nanog*, (c) *oct3/4*, and (d) *sox2* genes expression analyzed by real-time RT-PCR of expanded CB-CD34^+^ cells cultured in 4F and 5F in KSR compared with unexpanded cells (D0). The glyceraldehyde-3-phosphate dehydrogenase (GAPDH) was used as a house keeping gene control. Data shown are mean ± SD of three experiments. *P < 0.001, **P < 0.02.

In fact, gene expressions of *c-myc*, *nanog*,* oct3/4*, and *sox2* normally serve as pluripotency and self-renewal gene markers of embryonic stem cells and subsequently other stem cells including HSCs. Therefore, we further examined whether our expanded CD34^+^-enriched population in both 4F KSR and 5F KSR conditions could maintain stemness genes or not. To this end, cells of both conditions were separately collected after 5 days of expansion and further analyzed transcripts of *c-myc*, *nanog*, *oct3/4,* and *sox2* by real-time RT-PCR. The data showed that all genes were detected in 4F KSR and 5F KSR conditions (Figure 5). Surprisingly, *c-myc*, *nanog,* and *oct3/4* were upregulated in cells collected from 5F KSR medium than those collected from 4F KSR culture and fresh isolated CD34^+^ cells (*P* < 0.02, Figures 5(a), 5(b), and 5(c)). Thus, expanded cells in both 4F KSR and 5F KSR can maintain their stemness by preserve and/or support pluripotency genes expression. Thus, these findings support the importance of Wnt1 in the acceleration of CD34^+^CD38^−^ cells proliferation in 5F KSR medium for 7 days, which maintains the HSPCs properties at both cellular and molecular levels.

## 4. Discussion

Hematopoietic stem cells can be obtained from several sources. The 3 main sources are bone marrow (BM), peripheral blood (PB), and umbilical cord blood (UCB). Among these, cord blood (CB) serves as the most powerful source for HSCs collection, especially with noninvasive collection procedure. In addition, the CD34^+^ population containing HSCs isolated from CB exhibits superior advantages than those provided in BM and PB in many aspects, such as exhibiting lowest HLA antigens and containing highest potential of proliferation. These characteristics are crucial for achievement of hematopoietic stem cell transplantation [2]. Although the proportion of CD34^+^ cells is enriched in the CB, the cell quantity available in single unit is insufficient for autologous transplantation. Base on this regard, expansion technology is needed in order to obtain high yield of CD34^+^CD38^−^ cells and maintain their stemness and their ability to differentiate into all blood cell types. In this work, we succeeded to generate expansion culture condition that yield significant increase of CD34^+^CD38^−^ and CD133^+^CD38^−^ cells in serum-free medium supplemented with cytokine cocktail. 

The CD34^+^CD38^−^ cells represent primitive HSC population which is very crucial for blood cell transplantation applications. The CD133^+^CD38^−^ subpopulation appearing in CD34^+^-enriched population also serves as a source of quiescent stem cell which contains *in vivo* repopulating function [26, 27]. In addition, it has been investigated that CB AC133^+^CD38^−^ is an improved marker that tracts and enriches for LTC-IC and SRC [28]. Therefore, successful expansion of both CD34^+^CD38^−^ and CD133^+^CD38^−^ cells is a keystone for not only ability to overcome an insufficiency quantity of the cells but also the improvement of the stem cell transplantation. Interestingly, we observed the large amount of CD34^+^CD38^−^ cells (~18.5 folds) in 4 F KSR medium after 7 days of culture. More strikingly, in support of Wnt1 in the culture as 5F KSR medium, the higher significant increase of CD34^+^CD38^−^ cells was obtained (~24.3 folds) after 7 days of culture. These investigations showed that Wnt1 is a stimulator for the expansion of HSCs cells. The folds expansion of CB-CD34^+^CD38^−^ cells proliferation in this study showed greater achievement than previous study by Mishima and colleagues who obtained about 7-fold expansion by culturing the cells in cytokine combination of SCF, TPO, Flt3-L, IL-3, and IL-6 and in the presence of osteoblast-differentiated MSC feeder cells [16]. However, expansion of HSCs without feeder cells is more easy procedure to handle, very convenient to perform in large scale, and there is no problem in hematopoietic cells' attachment to the feeder cells. Additionally, we found that the number of CD34^+^CD38^−^ cells in serum-free medium was higher than those in FBS-containing medium. Therefore, the utility of serum-free conditions (4F KSR and 5F KSR) can reduce the risk of cross-contamination carried out by animal products and exhibit higher efficiency of CD34^+^CD38^−^ and CD133^+^CD38^−^ cells expansion in comparison with serum-containing media (4F cIMDM and 5F cIMDM). Furthermore, our data of 4F KSR and 5F KSR also indicated the lower quantity of CD34^−^CD38^+^ progenitor cells than those in 4F cIMDM and 5F cIMEM. The CD34^−^CD38^+^ or coexpression of CD34 and CD38 are more committed progenitor cells which may result in less effective in transplantation, while the more primitive HSC function is found to be enriched in CD34^+^CD38^−^ population [15]. These findings suggest that 4F KSR and 5F KSR are more appropriate culture conditions for HSPCs expansion and able to maintain CD34^+^ population than 4F cIMDM and 5F cIMEM culture conditions. Altogether, by comparison of all 4 conditions in this work, the efficiency of culture conditions for CB-CD34^+^CD38^−^ and CD133^+^CD38^−^ HSPCs expansion can be arranged in order from high to low efficiency as 5F KSR, 4F KSR, 5F cIMDM, and 4F cIMDM, respectively.

An* ex vivo* expansion of CD34^+^ cells using various cytokine cocktails is an alternative approach to overcome the limitation and has been developed largely by many research groups. The combination of cytokine factors: SCF, Flt3-L, TPO, and IL-6 has been widely used for *ex vivo* expansion of CD34^+^ cells in medium-containing serum [29–31]. However, prolonged culture resulted in an increasing of more committed progenitor cells which may dilute the number of true HSPCs and reduce the efficiency of engraftment. We also found the same phenomenon of high-yield progenitor cells in CB-CD34^+^ cells cultured in 4F cIMDM and 5F cIMDM serum containing conditions even in a short period of time as 7 days. Therefore, serum should be eliminated from the stem cell culture. It has been reported the use of SCF, Flt3-L, TPO, and IL-6/sIL-6R for HSPC expansion in serum-free condition and the capacity to increase NOD/SCID-repopulating cells expansion [32]. In addition, Seet's team showed that the treatment with valproic acid in serum-free condition for 7 days could enhance around 2-fold expansion of CD45^+^34^+^ progenitor cells [33]. These studies, however, observed the population of more committed HSPCs rather than primitive HSPCs. 

In fact, Wnt signals are activated through the canonical pathway for cell fate determination. Their role in hematopoiesis has been identified as a growth factor for the development of hematopoietic stem cells. Wnt1 has been suggested to play a role in the differentiation between human ESCs and hematoendothelial cells [34]. Previous studies have reported the significance of Wnt family signaling proteins in the expansion and self-renewal of HSPCs [22, 35]; however, Wnt1 has never been studied in CB-HSCs expansion before. Here, we demonstrated for the first time that Wnt1 effectively supported the CB-CD34^+^CD38^−^ and CD133^+^CD38^−^ cells' expansion. This effect was more potent in serum-free medium than serum-containing medium. In addition, Wnt1 supplementation in serum-free medium with Flt3-L, SCF, TPO and IL-6 (5F KSR) was also able to maintain the stemness property of expanded cells without affecting the differentiation capacity of hematopoietic progenitors. The 5F KSR-expanded cells could give rise to all blood cell types in the presence of suitable growth factors for each blood cell lineage differentiation. In contrast, cells were cultured in 4F KSR without Wnt 1; the expansion potential of HSCs was declined. Thus, this data confirms the stimulatory activity of Wnt1 on CB-HSCs proliferation. 

Generally, Wnt signaling pathway is mediated in the regulation of stem cell fate and maintenance of mouse ESCs and hESCs in undifferentiated state [34, 36]. Wnt3a, a canonical Wnt pathway activator, was found to promote short-term multilineage reconstitution of dormant c-kit cells [37]. Activation of Wnt/*β*-catenin signaling pathway can expand HSCs and plays a role in HSCs self-renewal. However, constitutive activation of *β*-catenin could abrogate HSCs differentiation and HSCs reconstitution *in vivo* [38]. Therefore, Wnt1 may exert function as the upregulator in CB-HSCs proliferation via the same pathway as Wnt3a or Wnt/*β*-catenin signaling pathway. It has been reported that conditioned medium collected from 293T transfected with Wnt1, Wnt5a, or Wnt10b could enhance fetal liver AA4^+^Sca^+^kit^+^ cells (murine HSCs) expansion [39]. The activation of Wnt pathway was also found in relation to Notch signaling, which is important in the early development of hematopoiesis [40]. Wnt-mediated maintenance of undifferentiated HSCs required the integration Notch signaling to inhibit differentiation. Thus, it would be suggested that Wnt1 may mediate the upregulation of Notch signaling pathway in expanded CB-HSCs and maintain stemness of the cells. Further process is required to clarify the role of Wnt1 protein in interaction with Notch signaling in the regulation of HSCs self-renewal. 

In the present study, we also investigated whether enhancement of proliferation of CD34^+^ cells or HSPCs by 4F KSR and 5F KSR would affect the pluripotency and self-renewal activity. In cellular level, we demonstrated that the expanded cells have ability to produce clonogenic progenitor cells: CFU-GEMM, CFU-GM, BFU-E, and CFU-M similar to the fresh isolated CD34^+^ cells (Figure 4(a)). In addition, liquid culture differentiation assay indicated the capability of HSPCs in both 4F KSR and 5F KSR to generate more mature blood cells (Figures 4(c) and 4(d)). These findings support the achievement of repopulating capacity of expanded HSPCs population. In molecular level, we also demonstrated that expanded HSPCs from both 4F KSR and 5F KSR expressed key pluripotency and self-renewal genes of *c-myc*, *nanog*, *oct3/4* and *sox2*. Oct3/4, one key transcription factor mediated to the pluripotency maintenance of ES cells, was found to regulate *sox2* expression in ES cells which then activated Oct-Sox enhancers to control expression of *nanog* and even of *oct3/4* and *sox2* themselves  [41]. Therefore, our data revealed mRNA expression of transcription factors oct3/4, nanog, and sox2 which mediate maintenance of pluripotency and self-renewal of ESCs [42, 43]. Our results also showed the resemblance expression of these genes in HSPCs as in fresh isolated CD34^+^ cells. Thus, these suggested that 4F KSR and 5F KSR cocktails could stabilize the expression of pluripotency and self-renewal genes which are crucial characteristics of stem cells. 

HSCs transplantation has been used for the treatment of hematopoietic disorders, solid malignant tumors [44, 45], and nonhematopoietic diseases as currently addressed in clinical trials in many countries around the world. Besides the property of expanded HSPCs in sustainment of the stem cell pool in hematopoietic system of transplanted patient, commitment to give rise to blood cells is also essential for immunological functions. After high-dose chemotherapy in cancer patients, neutropenia normally occurs. Myeloid lineages which play roles in the innate immune responses can protect against bacterial infections in the initial phase of transplantation. Our result is consistent with previous observation that *ex vivo* expansion of CD34^+^ cells predominantly committed into myeloid progenitor cells with a negligible expansion into lymphoid lineage [46]. Thus, the naturally myeloid/erythroid commitment by *ex vivo* culture may enhance engraftment efficiency and reduce mortality rate in clinical studies. Recently, Phase I clinical trial demonstrated that the utilization of the *ex vivo* expanded CB-CD34^+^ cells along with nonmanipulating CB unit facilitated myeloid engraftment rate in patients [47]. This data, therefore, highlights the importance of expanded CB unit for therapeutic purpose even though the real mechanism of supportive engraftment between nonmanipulating and manipulate one has not been explored.

In conclusion, this work demonstrates that in the presence of the same cytokine cocktail, the serum-free medium is a better option than utilizing serum-containing medium for CD34^+^CD38^−^ and other HSPCs expansion, not only less animal product contamination but higher expansion efficiency of the cells. In addition, Wnt1 can synergize SCF, Flt-3L, TPO, and IL-6 in serum-free medium (5F KSR) to stimulate CD34^+^CD38^−^ and CD133^+^CD38^−^ HSPCs proliferation. The advantage of the presence of both populations simultaneously is that they can synergize and enhance the capacity of blood cells' reconstitution. Moreover, the utilization of human cytokines in the culture media is feasible, safe, and not complicated or at risk by the use of animal product system. Furthermore, this cocktail can maintain hematopoietic properties of HSPCs at both cellular and molecular levels. Finally, Wnt1 can stimulate the survival and proliferation of HSPCs, demonstrating that Wnt1 comprises a novel class of hematopoietic cell regulators. The implications of this work are future therapeutic value in cord blood transplantation for hematologic and nonhematologic diseases, blood bank/stem cell bank applications, and hematological studies. Development of higher efficiency expansion method will also be further explored.

## Figures and Tables

**Table 1:** Primer lists.

Primer	Forward primer (5′-3′)	Location	Reverse primer (5′-3′)	Location	Size (bp)	Reference sequence
*c-Myc *	TGGTCTTCCCCTACCCTCTCAAC	C.1148_1170	GATCCAGACTCTGACCTTTTGCC	C.1390_1412	265 [23]	NM_002467.4
*Oct3/4 *	CTCACCCTGGGGGTTCTATT	C.559_578	CTCCAGGTTGCCTCTCACTC	C.773_792	233 [23]	NM_002701.4
*Sox2 *	GCCCCCAGCAGACTTCACA	C.1299_1317	CTCCTCTTTTGCACCCCTCCCATTT	C.1143_1467	169 [23]	NM_003106.3
*Nanog *	GCTTGCCTTGCTTTGAAGCA	C.245_264	TTCTTGACCGGGACCTTGTC	C.481_500	256 [24]	NM_024865.2
*GAPDH *	AGCCACATCGCTCAGACACC	C.155_174	GTACTCAGCGGCCAGCATCG	C.438_456	302 [24]	NM_002046.4

**Table 2:** Folds expansion of CD34^+^CD38^−^, CD133^+^CD38^−^, and CD34^−^CD38^+^ cells under various cytokine culture conditions.

Culture conditions			Day	Fold expansion		
Name	Medium	Cytokines		CD34^+^CD38^−^	CD133^+^CD38^−^	CD34^−^CD38^+^
4F cIMDM	IMDM + FBS	Flt3-L, SCF, TPO, and IL-6	5	2.6 ± 1.1	0.7 ± 0.4	12.0 ± 1.4
			7	4.2 ± 1.3	3.1 ± 1.6	62.8 ± 5.0
5F cIMDM	IMDM + FBS	Flt3-L, SCF, TPO, IL-6, and Wnt1	5	3.9 ± 1.0	2.5 ± 1.2	9.3 ± 1.3
			7	6.6 ± 1.4	6.0 ± 2.6	89.7 ± 12.2***
4F KSR	IMDM + KSR	Flt3-L, SCF, TPO, and IL-6	5	6.3 ± 1.6	4.7 ± 2.0	7.6 ± 2.2
			7	18.5 ± 0.4*	11.5 ± 3.4***	20.1 ± 6.8*
5F KSR	IMDM + KSR	Flt3-L, SCF, TPO, IL-6, and Wnt1	5	5.9 ± 1.2	5.4 ± 2.2	8.4 ± 2.2
			7	24.3 ± 2.1^∗,∗∗^	12.3 ± 4.0***	13.0 ± 3.7*

Data represented as mean fold increasing in expansion of three independent CB sample ± SD expanded for 5 and 7 days subtracted with day 0 (*n* = 3). *P ≤ 0.001 versus 4F cIMDM and 5F cIMDM, **P = 0.005 versus 4F KSR, ***P < 0.05 versus 4F cIMDM on the same day of culture.

**Table 3:** Immunophenotype of subpopulation of expanded cells analyzed by flow cytometry at days 5 and 7.

Population	Fresh isolate	Day 5				Day 7			
		4F cIMDM	5F cIMDM	4F KSR	5F KSR	4F cIMDM	5F cIMDM	4F KSR	5F KSR
CD34^+^CD38^−^	7.8 ± 1.6	3.1 ± 0.5	5.1 ± 2.9	8.8 ± 3.4	6.8 ± 1.9	3.0 ± 2.5*	3.9 ± 4.0	13.4 ± 6.5	16.1 ± 3.9
CD133^+^CD38^−^	7.5 ± 4.2	0.7 ± 0.2	2.7 ± 2.2	5.6 ± 3.5	5.4 ± 5.1	1.8 ± 1.3	3.0 ± 3.7	6.6 ± 2.9	6.5 ± 1.9
CD34^−^CD38^+^	5.7 ± 0.9	11.1 ± 1.0	8.9 ± 1.9	7.8 ± 5.9	7.2 ± 4.8	34.2 ± 12.8*	39.3 ± 12.8*	9.6 ± 8.1**	5.7 ± 0.9

Data shown as mean ± SD values (*n* = 3).

*P < 0.05 versus 5F KSR, **P < 0.05 versus 5F cIMDM.
